# Draft genome sequence of *Raoultella terrigena* strain Ech2A causing soft rot on sweet pepper (*Capsicum annuum*)

**DOI:** 10.1128/mra.00159-24

**Published:** 2024-09-27

**Authors:** J. Sandoval-Gutiérrez, D. Cubero-Agüero, L. Brenes-Guillén, F. Galiano-Murillo, D. Vidaurre-Barahona, L. Uribe-Lorío

**Affiliations:** 1 School of Biological Sciences, Universidad Nacional of Costa Rica, Heredia, Costa Rica; 2 Center for Research in Cellular and Molecular Biology, Universidad de Costa Rica, San José, Costa Rica; 3 School of Agronomy, Universidad de Costa Rica, San José, Costa Rica; The University of Arizona, Tucson, Arizona, USA

**Keywords:** phytopathogen, pectinolytic enzymes, Enterobacterales

## Abstract

We report the draft genome sequence of *Raoultella terrigena* strain Ech2A causing soft rot on pepper. To verify pathogenicity, Koch’s postulates were performed on sweet pepper. Genes encoding pectinolytic enzymes were found in the genome.

## ANNOUNCEMENT

Bacterial soft rot is caused by pathogens that can degrade pectin, a component of the cell wall of plant tissues, including crops such as sweet pepper (*Capsicum annuum*). While *Dickeya* and *Pectobacterium* are the most widely studied soft-rot bacterial pathogens, other genus species also cause soft rot on plant organs, yet little information is available regarding these pathogens (1). Sweet peppers exhibiting symptoms of soft rot were collected from plants between 3 and 4 months of maturity in Oreamuno, Cartago, Costa Rica (9.86916667,–83.90333333). The disease symptoms observed on the pepper fruits and the details of the bacterial isolation process were reported by Cubero-Agüero and colleagues (2). The symptomatic tissue edges were excised and macerated in 10 mL of 0.85% NaCl solution. The resulting suspension was streaked onto MacConkey agar and incubated at 30°C for 24 hours. Subsequently, different morphotypes were tested for pectinolytic activity on crystal violet pectate medium (3). DNA was purified using Macherey-Nagel NucleoSpin Microbial DNA Extraction Kit according to the manufacturer’s instructions. The isolate with the highest pectolytic activity was identified by polymerase chain reaction amplification of the region 16S rRNA, using the universal primer set of 27F/907R (4). Strain Ech2A showed 98.13% similarity to *Raoultella terrigena* JCM1687 (type strain) (5), as described in reference (2). To verify the pathogenicity, Ech2A was cultivated on nutrient agar (Oxoid) at 30°C for 24 hours. Additionally, Koch’s postulates were tested by injecting 50 µL of bacterial suspension (9 × 10^8^ CFU/mL) into three different sections of healthy pepper fruits, which were then incubated at 30°C for 5 days until soft rot symptoms were observed. Sterile distilled water was used as a negative control (2).

For genomic analysis, total DNA was processed by using a NexteraXT DNA Library Prep Kit and MiSeq paired-end sequencing (MiSeq Reagent Kit v2, 2 × 250 bp, Illumina, Inc.) in the Center for Research in Cellular and Molecular Biology of the Universidad de Costa Rica. Bioinformatic analyses were performed using the Kabré Supercomputer from the National High Technology Center, Costa Rica. Default parameters were used for all software unless otherwise specified. The sequenced reads were filtered using Trimmomatic version 0.36 (6), SLIDINGWINDOW:4:15, and MINLEN of 100 bp, resulting in 426,968 reads. *De novo* genome assembly was performed using SPAdes version 3.11.0 (7). Prokka version 1.12 (8) was used to automatically generate annotations. The publicly accessible genome at GenBank was annotated using NCBI’s Prokaryotic Genome Annotation Pipeline (9). To assess the average nucleotide identity (ANI), we employed an ANI calculator to compare genomes (10). To estimate the completeness and redundancy, we used CheckM (11). The draft genome of *Raoultella terrigena* strain Ech2A consists of 452 contigs totaling 5,504,929 bp, with an *N*50 value of 23,847, a GC content of 58%, and a genome coverage of 10×. The genome contains 70 tRNA, 5,272 and 187 coding and non-coding sequences, respectively. The completeness is 98.9% and the redundancy is 0.83%. The genomic similarity between *Raoultella terrigena* NBRC 14941 and Ech2A is 97.10%. The following genes were found in the Prokka annotation: endo-polygalacturonase (*pehA*) and exo-pectate lyase (*pelX*) genes, which are involved in the degradation of pectin and present in bacterial plant pathogens such as the *Pectobacterium* genus (12).

## Data Availability

Amplified product was sequenced and deposited in NCBI (GenBank accession MN386196). The genome sequence of *Raoultella terrigena* strain Ech2A was deposited in DDBJ/ENA/GenBank under accession number GCA_030078455.1 and BioProject accession number PRJNA898399. The raw data are available under the SRA accession number SRR29857959.
